# Travelling Waves of a Delayed SIR Epidemic Model with Nonlinear Incidence Rate and Spatial Diffusion

**DOI:** 10.1371/journal.pone.0021128

**Published:** 2011-06-24

**Authors:** Jing Yang, Siyang Liang, Yi Zhang

**Affiliations:** 1 Department of Physiology, Hebei Medical University, Shijiazhuang, People’s Republic of China; 2 Beijing Institute of Technology, Beijing, People’s Republic of China; University of Swansea, United Kingdom

## Abstract

This paper is concerned with the existence of travlelling waves to a SIR epidemic model with nonlinear incidence rate, spatial diffusion and time delay. By analyzing the corresponding characteristic equations, the local stability of a disease-free steady state and an endemic steady state to this system under homogeneous Neumann boundary conditions is discussed. By using the cross iteration method and the Schauder's fixed point theorem, we reduce the existence of travelling waves to the existence of a pair of upper-lower solutions. By constructing a pair of upper-lower solutions, we derive the existence of a travelling wave connecting the disease-free steady state and the endemic steady state. Numerical simulations are carried out to illustrate the main results.

## Introduction

Let 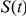 represent the number of individuals who are susceptible to the disease, that is, who are not yet infected at time 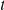; 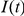 represent the number of infected individuals who are infectious and are able to spread the disease by contact with susceptible individuals, and 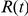 represent the number of individuals who have been infected and then removed from the possibility of being infected again or of spreading at time 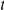. In [1], Cooke formulated a SIR model with time delay effect by assuming that the force of infection at time t is given by 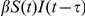, where 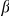 is the average number of contacts per infective per day and 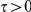 is a fixed time during which the infectious agents develop in the vector, and it is only after that time that the infected vector can infect a susceptible human. Cooke considered the following model
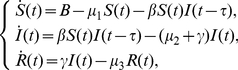
(0.1)where parameters 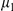, 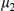, 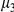 are positive constants representing the death rates of susceptibles, infectives, and recovered, respectively. It is natural biologically to assume that 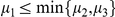. The parameters 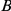 and 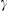 are positive constants representing the birth rate of the population and the recovery rate of infectives, respectively. Much attention has been paid to the analysis of the stability of the disease free equilibrium and the endemic equilibrium of system (1.1) (see, for example, [2], [3], [4], [5]).

Incidence rate plays an important role in the modelling of epidemic dynamics. It has been suggested by several authors that the disease transmission process may have a nonlinear incidence rate. In [6], Xu and Ma considered the following SIR epidemic model with time delay and nonlinear incidence rate
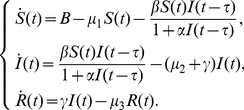
(0.2)By analyzing the corresponding characteristic equations, they studied the local stability of an endemic equilibrium and a disease free equilibrium. It was proved that if the basic reproductive number 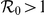, the system was permanent. By comparison arguments, it was shown that if 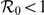, the disease free equilibrium was globally asymptotically stable. If 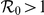, by means of an iteration technique and Lyapunov functional technique, respectively, sufficient conditions were obtained for the global asymptotic stability of the endemic equilibrium.

We note that the spatial content of the environment has been ignored in the models aforementioned. The models have been traditionally formulated in relation to the time evolution of uniform population distributions in the habitat and are as such governed by ordinary differential equations. However, due to the large mobility of people within a country or even worldwide, spatially uniform models are not sufficient to give a realistic picture of disease diffusion. For this reason, the spatial effects can not be neglected in studying the spread of epidemics. Noble [7] applied reaction-diffusion theory to describe the spread of plague through Europe in the mid-14th century. By using the linear theory of semigroups, Saccomandi [8] investigated the existence and uniqueness of the solution for an SIR model with spatial inhomogeneity, nonlocal interactions, and an open population. In recent times, many investigators have introduced population movements into related equations for epidemiological modeling and simulations in efforts to understand the most basic features of spatially distributed interactions (see, for example, [9], [10], [11], [12], [13]).

Motivated by the work of Xu and Ma [6] and Noble [7], in the present paper, we are concerned with the following delayed SIR epidemic model with nonlinear incidence rate and spatial diffusion
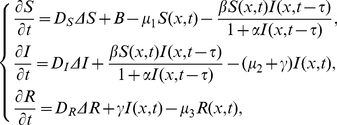
(0.3)with initial conditions
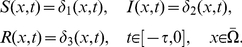
(0.4)In problem (3)-(4), the positive constants 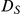, 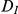 and 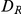 denote the corresponding diffusion rates for the susceptible, infected and removed populations, respectively; 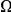 is a bounded domain in 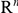 with smooth boundary 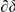. The functions 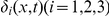 are nonnegative and Hölder continuous and satisfy 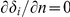 in 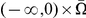. In this paper, we assume that 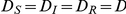.

In the biological context, it is important to analyze the epidemic waves which is described by traveling wave solutions propagating with a certain speed. In this paper, we are interested in the existence of travelling wave solutions to SIRS epidemic model (0.3). The main tool to study the existence of travelling wave solutions for the reaction-diffusion equations with delays is the sub- and supersolution technique due to Atkinson and Reuter [14]. Wu and Zou [15], [16] studied the existence of travelling wavefronts for delayed reaction-diffusion systems with reaction terms satisfying the so-called quasi-monotonicity or exponential quasi-monotonicity conditions, where the well-known monotone iteration techniques for elliptic systems with advanced arguments [17], [18] were used. Ge and He [19] and Ge et al. [20] used the iteration technique developed by Wu and Zou [15] to investigate the existence of travelling wave solutions for two-species predator-prey system with diffusion terms and stage structure, respectively. However, we note that the nonlinear reaction terms of system (0.3) do not satisfy either the quasi-monotonicity or the exponential quasi-monotonicity conditions. Therefore, the method of upper-lower solutions and its associated monotone iteration scheme developed by Wu and Zou [15], [16] can not be used to study the existence of travelling wave solutions to system (0.3). Recently, by constructing a pair of desirable upper-lower solutions, Huang and Zou [21] got a subset, and employed the Schauder's fixed point theorem in this subset to investigate the existence of travelling wave solutions of a class of delayed reaction diffusion systems with two equations in which the nonlinear reaction terms satisfy the partial quasi-monotonicity and partial exponential quasi-monotonicity, respectively. Li et al. [22] investigated the existence of travelling wave solutions of a class of delayed reaction diffusion systems with two equations in which the reaction terms satisfy weak quasi-monotonicity and weak exponential quasi-monotonicity conditions, respectively. Sazonov et al. [23], [24] studied the problems of travelling waves in the SIR model. Clearly, all the results above can not directly be applied to a system with more than three equations. Therefore, it remains important and challenging to study the existence of travelling wave solutions for delayed reaction diffusion systems with more than three equations in which the nonlinear reaction terms do not satisfy either the quasi-monotonicity or the exponential quasi-monotonicity conditions.

## Methods

### Preliminaries

Throughout this paper, we adopt the usual notations for the standard ordering in 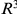. Thus, for 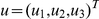 and 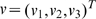, we denote 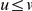 if 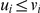, 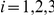; 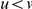 if 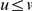 but 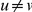; and 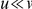 if 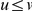 but 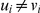, 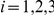. If 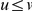, we also denote 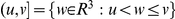, 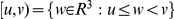, and 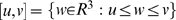. We use 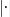 to denote the Euclidean norm in 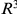 and 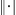 to denote the supremum norm in 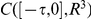.

Before proving the existence of travelling wave solutions to system (0.3), we first investigate the following general delayed reaction-diffusion system:
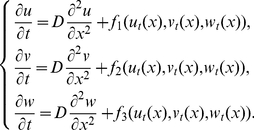
(0.5)


On substituting 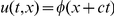, 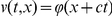, 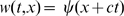 and denote the travelling wave coordinate 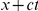 still by 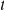, we derive from (0.5) that
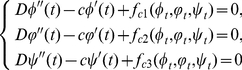
(0.6)satisfying the following partial quasi-monotonicity conditions 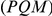:


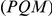
 There exist three positive constants 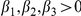 such that
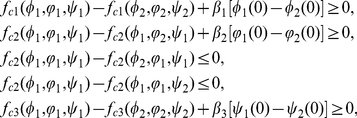
(0.7)where 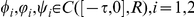, with 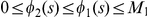, 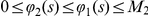, 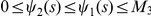, 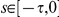, 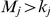 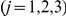 are positive constants, 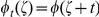, 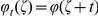, 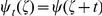, and the functions 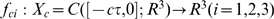 are defined by
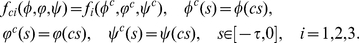
If, for some 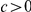, system (5) has a solution defined on 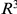 satisfying
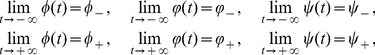
(0.8)where 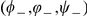 and 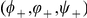 are steady states of (0.5). Then 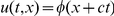, 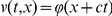, 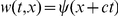 is called a travelling wave solution of system (0.5) with speed 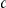. Without loss of generality, we can assume 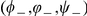 = (0,0,0) and 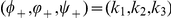, and seek for travelling wave solution connecting these two steady states.

Corresponding to (0.5), we make the following hypotheses:

(*A*1) 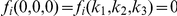, 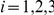.

(*A2*) There exist three positive constants 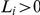 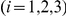 such that
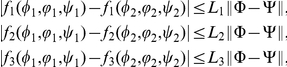
for 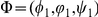, 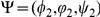 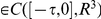 with 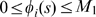, 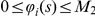, 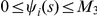, 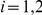.

In the next section, we will apply the Schauder's fixed point theorem, which requires the continuity of the operator under consideration. For this purpose, we need to introduce a topology in 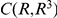. Let 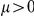 and equipped 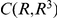 with the exponential decay norm defined by
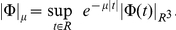
Define
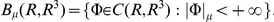
Then it is easy to check that 
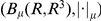
 is a Banach space.

We look for travelling wave solutions to system (0.5) in the following profile set:
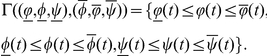
Obviously, 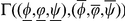 is non-empty, convex, closed and bounded.

We also need the following definition of upper and lower solutions to system (0.5).

#### Definition 0.1

*A pair of continuous functions 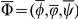 and 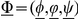 are called a pair of upper-lower solutions of system *(0.5) *if 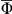 and 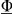 are twice differentiable almost everywhere in 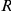 and they are essentially bounded on 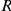, and there hold*

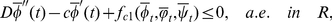
(0.9)

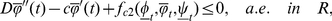
(0.10)

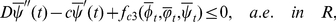
(0.11)and
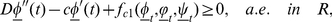
(0.12)

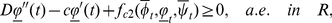
(0.13)

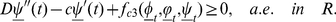
(0.14)


Unlike the standard upper and lower solutions defined in2_3_ [15], 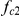 is evaluated in a cross iteration scheme given in (0.10) and (0.13).

### Local stability

In this section, by analyzing the corresponding characteristic equations, we discuss the local stability of an endemic equilibrium and a disease-free equilibrium of system (0.3) with the initial conditions (0.4) and the homogeneous Neumann boundary conditions
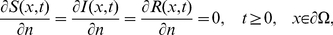
(0.15)respectively, where 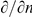 denotes the outward normal derivative on 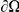, the homogeneous Neumann boundary conditions imply that the populations do not move across the boundary 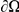.

System (0.3) always has a disease-free steady state 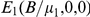. Further, if 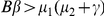, then system (0.3) has a unique endemic steady state 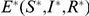, where
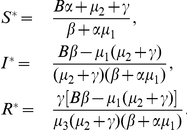



Let
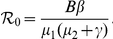


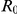
 is called the basic reproductive number (sometimes called basic reproductive rate or basic reproductive ratio) of system (0.3), which describes the average number of newly infected cells generated from one infected cell at the beginning of the infectious process. It is easy to show that if 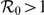, the endemic steady state 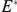 exists; if 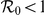, 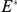 is not feasible.

Let 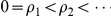 be the eigenvalues of the operator 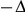 on 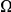 with the homogeneous Neumann boundary conditions, and 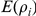 be the eigenspace corresponding to 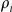 in 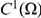. Let 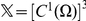, 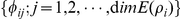 be an orthonormal basis of 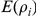, and 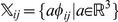. Then
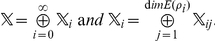
Let 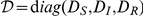, 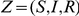, 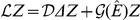, where
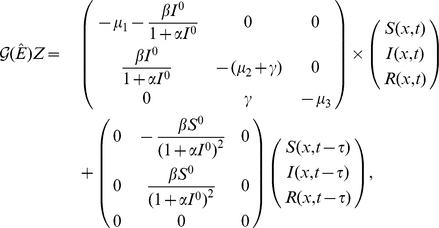
and 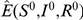 represents any feasible uniform steady state of system (0.3). The linearization of system (0.3) at 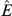 is of the form 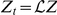. For each 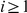, 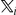 is invariant under the operator 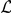, and 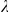 is an eigenvalue of 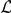 if and only if it is an eigenvalue of the matrix 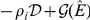 for some 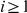, in which case, there is an eigenvector in 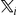.

The characteristic equation of 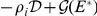 takes the form
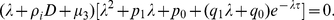
(0.16)where
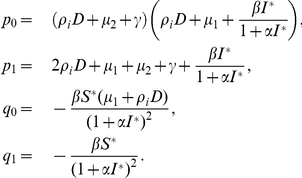
Clearly, for any 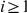, Eq. (0.16) always has one negative real root 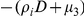. Its other roots are determined by the following equation
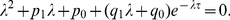
(0.17)When 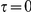, Eq. (0.17) becomes
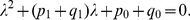
(0.18)It is readily seen that if 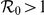, then
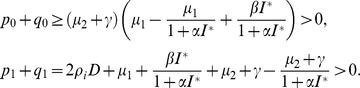
Hence, if 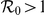, the endemic steady state 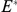 of system (0.3) is locally stable when 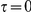.

If 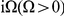 is a solution of (0.16), separating real and imaginary parts, we derive that
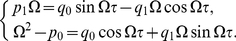
(0.19)


Squaring and adding the two equations of (0.19), it follows that
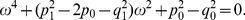
(0.20)Let 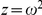, then Eq. (0.20) becomes
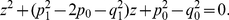
(0.21)By calculation it follows that for all 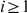


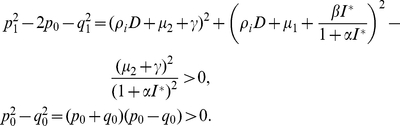
Hence, we can know that Eq. (0.21) has no positive roots. Therefore, if 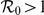, 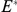 is locally asymptotically stable for all 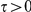.

The characteristic equation of 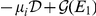 is of the form
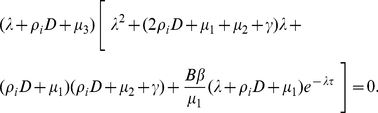
(0.22)Clearly, for any 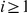, Eq. (0.22) always has a negative real root 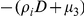. All other roots are given by the roots of equation
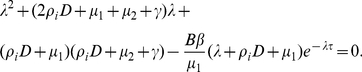
(0.23)Let
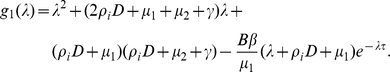
If 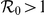, it is readily seen that for 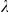 real,
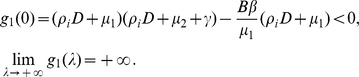
Hence, when 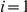, (23) has a positive real root. Therefore, there is a characteristic root 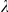 with positive real part in the spectrum of 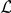. Accordingly, if 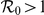, 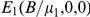 is unstable.

We therefore obtain the following results.

#### Theorem 0.1

*For system* (0.3) *with initial conditions* (0.4) *and homogeneous Neumann boundary conditions* (0.15), *we have*

(*i*) If 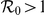, the disease-free steady state 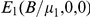 is unstable; if 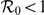, 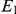 is locally asymptotically stable.

(*ii*) Let 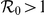, the endemic steady state 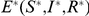 is asymptotically stable for all 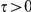.

## Results

### Existence of travelling waves for system (5)

In this section, we study the existence of travelling wave solutions to system (0.5) with nonlinear reaction terms satisfying 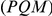.

We assume that a pair of upper-lower solutions 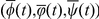 and 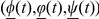 are given such that

(*P*1) 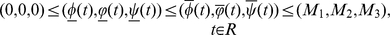,

(*P2*) 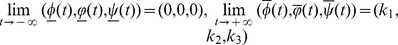.

For the constants 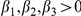 in 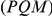, define 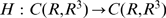 by
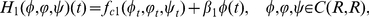
(0.24)

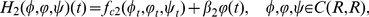
(0.25)

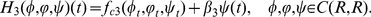
(0.26)


The operators 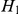, 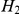 and 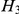 admit the following properties:

#### Lemma 0.1

*Assume that 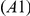 and 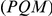 hold, then*

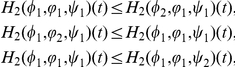
for 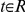 with 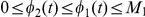, 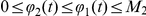, 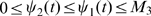.

Proof. By 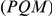, direct calculation shows that
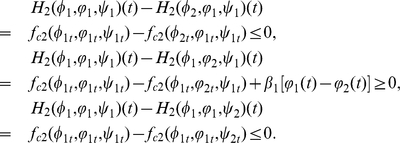
This completes the proof.

#### Lemma 0.2

*([15]) Assume that 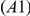 and 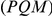 hold. Then for any* 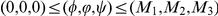, *we have*

(i) 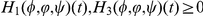, 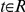.

(ii) 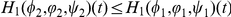 and 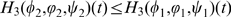 for 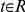 with 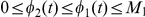, 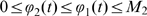, 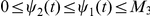.

In terms of 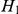, 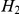 and 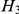, system (0.34) can be rewritten as
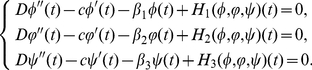
(0.27)Define
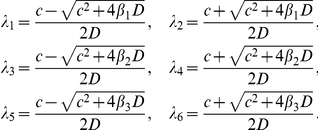
Let
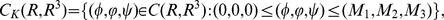
and define 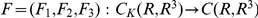 by
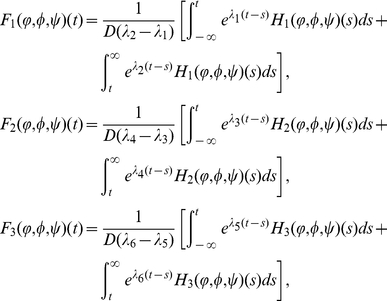
for 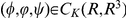. It is easy to see that 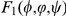, 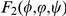 and 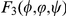 satisfy
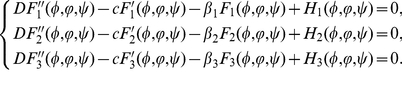
(0.28)


Corresponding to Lemmas 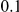 and 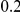, we have the following result for 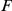.

#### Lemma 0.3

*Assume that 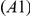 and 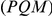 hold. For any 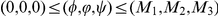, we have 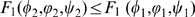, 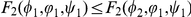, 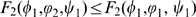, 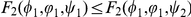, 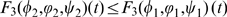 for 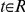 with 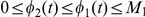, 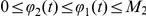, 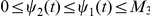.*

We next verify the continuity of 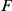.

#### Lemma 0.4

*Assume* 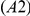 *holds*, *then* 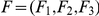 *is continuous with respective to the norm* 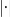 in 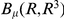.

Proof. For any fixed 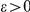, let 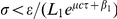, then for 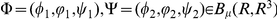 with
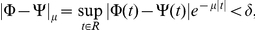
direct calculation shows that
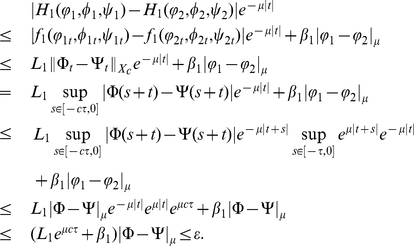
For 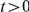, we see that
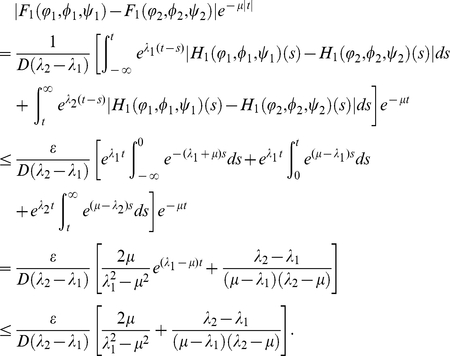
Similarly, for 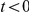, we have
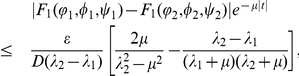
which implies that 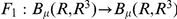 is continuous with respect to the norm 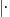 in 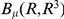.

By using a similar argument as above, it can be shown that 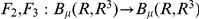 are continuous. Thus, we obtain that 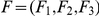 is continuous with respect to the norm 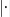 in 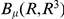. This completes the proof.

#### Lemma 0.5

*Assume that 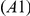 and 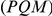 hold, then*

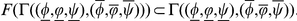
Proof. For any 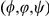 with 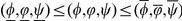, it follows from Lemma 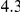 that
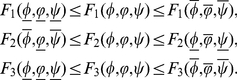
(0.29)


By the definition of upper-lower solutions, we have
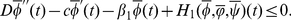
(0.30)Choosing 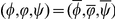 in the first equation of (0.28), and denoting 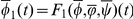, we get
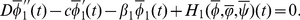
(0.31)Setting 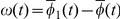 and combining (0.30) and (0.31), we have
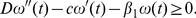
(0.32)Repeating the proof of Lemma 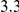 in Wu and Zou [15] shows that 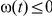, which implies that 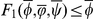.

By a similar argument, we know that 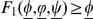, 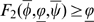, 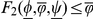, 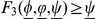, 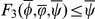, then 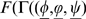. This completes the proof.

#### Lemma 0.6

*Assume 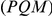 holds, then 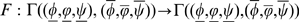 is compact.*

Proof. Noting that
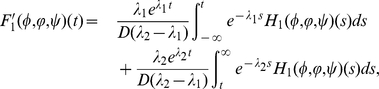
it follows from in Lemma 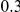 that 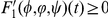. By 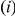 in Lemma 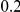 and the fact that 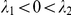, we have
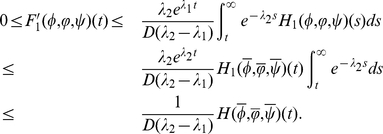
Hence, 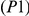 implies that there exists a constant 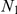 such that 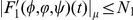.

For any 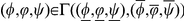,
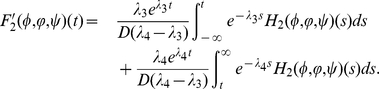
Thus, we have
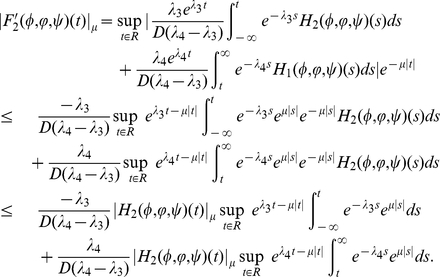
If 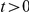, we get
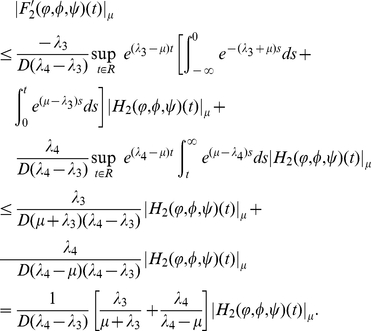
If 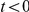, we obtain
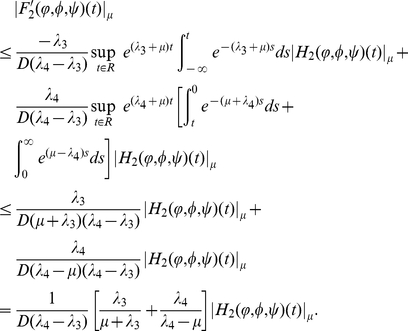



Noting that 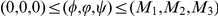, it follows from Lemma 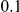 that 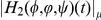 is bounded by a positive number. Therefore, there exists a constant 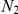 such that 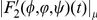 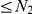.

Similar to the proof of 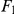, we have that there exists a constant 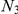 such that 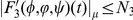.

The above estimate for 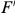 shows that 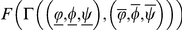 is equicontinuous. It follows from the proof of Lemma 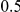 that 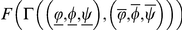 is uniformly bounded.

Next, we define
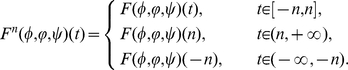
Then, for each 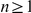, 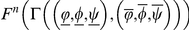 is also equicontinuous and uniformly bounded. Now, in the interval 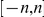, it follows from Ascoli-Arzela Theorem that 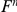 is compact. On the other hand, 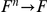 in 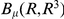 as 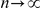, since
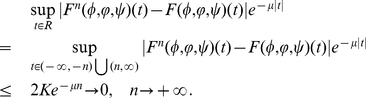
By Proposition 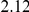 in [25], we have that 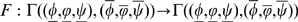 is compact. This completes the proof.

#### Theorem 0.2

*Assume that 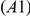, 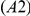 and 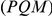 hold. Suppose there is a pair of upper-lower solutions 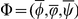 and* 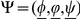 *for* (0.5) *satisfying 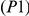 and 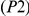. Then, system *(0.5) *has a travelling wave solution.*

Proof. Combining Lemmas 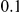-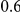 with the Schauder's fixed point theorem, we know that there exists a fixed point 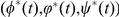 of 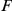 in 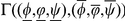, which gives a solution to (0.5).

In order to prove that the solution is a travelling wave solution, we need to verify the asymptotic boundary conditions (0.8).

By 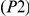 and the fact that
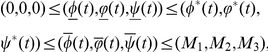
we get that
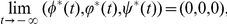
and
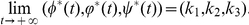
Therefore, the fixed point 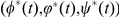 satisfies the asymptotic boundary conditions (0.8). This completes the proof.

### Existence of travelling waves for system (0.3)

In this section, we use the results developed in Section 4 to study the existence of travelling wave solutions to system (0.3).

Denoting 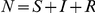, then system (0.3) is equivalent to the following system
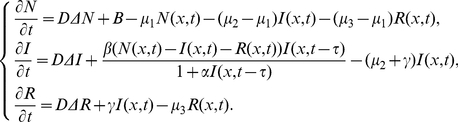
(0.33)


By making change of variables 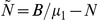, 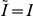, 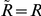 and dropping the tildes, system (0.33) becomes
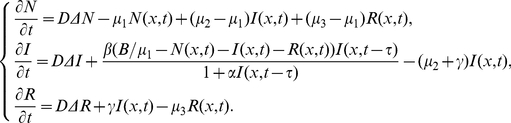
(0.34)


It is easy to show that if 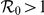, system (0.34) has two steady states 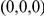 and 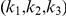, where
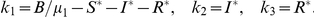



On substituting 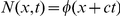, 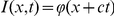, 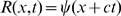 and denote the travelling wave coordinate 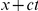 still by 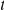, we derive from (34) that
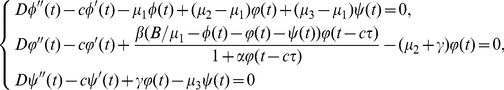
(0.35)satisfying the following asymptotic boundary conditions
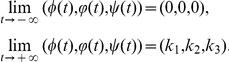



#### Lemma 0.7

*The nonlinear reaction terms of system *(0.34) *satisfy* 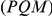.

Proof. For any 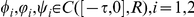, with 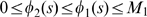, 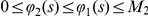, 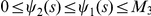, 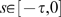, we have
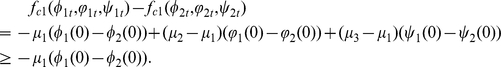
Let 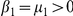. We derive that 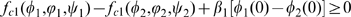.

For 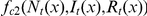, it follows that
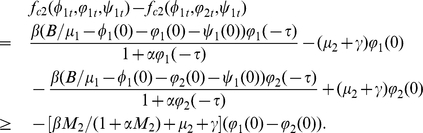
Let 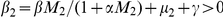. We know that 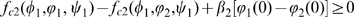.
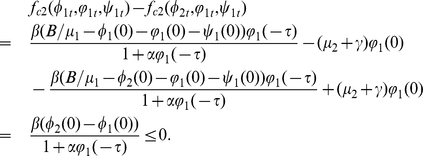


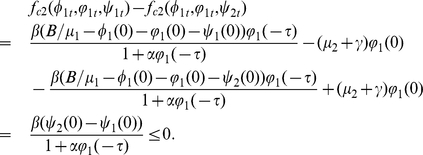
For 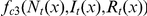, we have
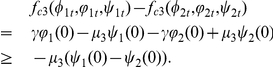
Let 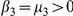. We obtain 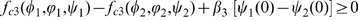. This completes the proof.

Let
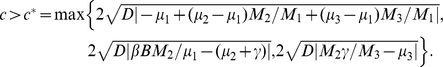
There exist 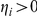 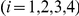 such that
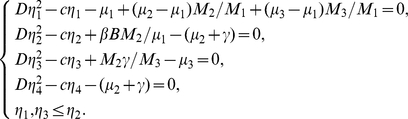
We can find that there exist 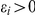 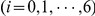 satisfying
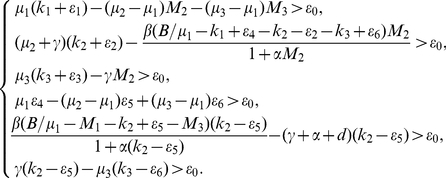
(0.36)


For the above constants, suitable constants 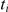 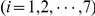 and 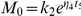 satisfying 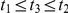, 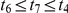 and 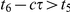, we define the continuous functions 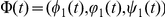 and 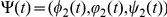 as follows
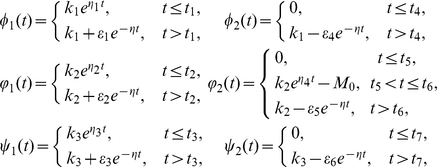
where 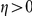 is a constant to be chosen later. It is easy to know that 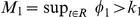, 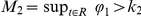, 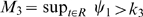, 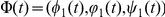 and 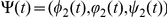 satisfy (36) and the following conditions:

(P1) 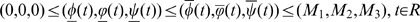,

(P2) 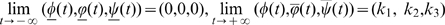.

#### Lemma 0.8


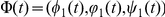
 *is an upper solution of system* (0.35).

Proof. If 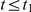, 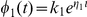, 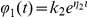 and 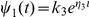. It follows that
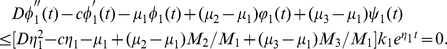
If 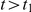, 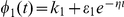. We have
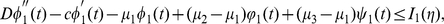
where
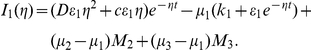
It follows from (0.36) that 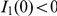 and there exists 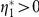 such that 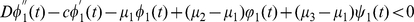 for all 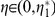.

If 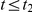, 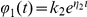. It follows that
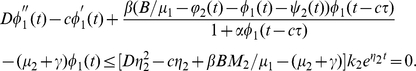
If 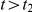, 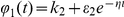. We get
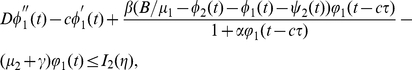
where
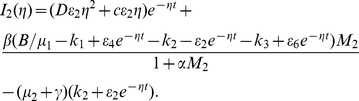
It follows from (0.36) that 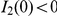 and there exists 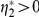 such that 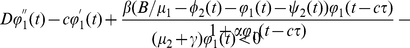 for all 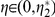.

If 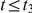, 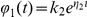 and 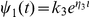. It follows that
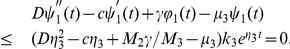
If 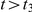, 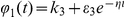. We get
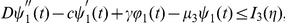
where
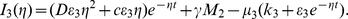
It follows from (0.36) that 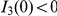 and there exists 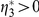 such that 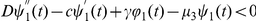 for all 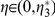.

Taking 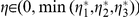, we see that the conclusion is true. This completes the proof.

#### Lemma 0.9


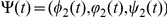
 *is a lower solution of system* (0.35).

Proof. If 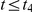, 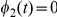. It follows that
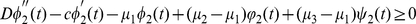
If 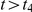, 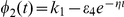 and 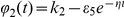. We have
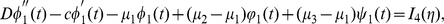
where
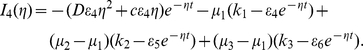
It follows from (0.36) that 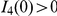 and there exists 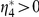 such that 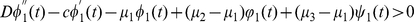 for all 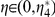.

If 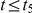, 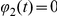. It follows that
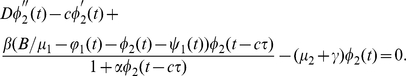
If 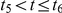, 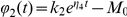. We get
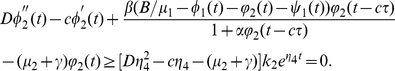
If 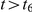, 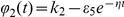. We know
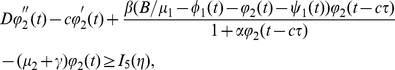
where
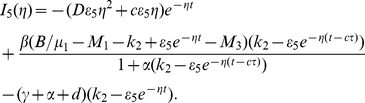
It follows from (0.36) that 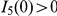 and there exists 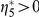 such that 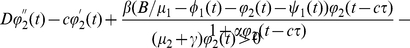 for all 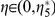.

If 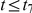, 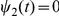. It follows that
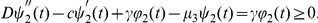
If 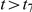, 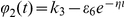. We get
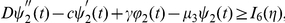
where
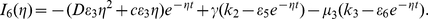
It follows from (0.36) that 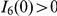 and there exists 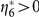 such that 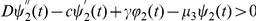 for all 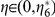.

Letting 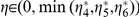, we see that our conclusion is true. This completes the proof.

Applying Lemmas 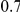-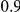, we know that if 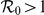, system (0.34) has a travelling wave solution with speed 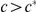 connecting the steady states 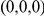 and 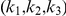. Accordingly, we have the following conclusion.

#### Theorem 0.3

*Let* 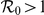. *For every* 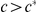, *regardless of the value of* 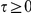, *system* (0.3) *always has a travelling wave solution with speed* 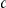 *connecting the uninfected steady state 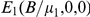 and the infected steady state* 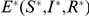.

#### Remark

The travelling wave solution in Theorem 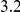 may not be monotonic. The fact is illustrated by the following numerical simulations.

### Numerical simulations

In this section, by using the classical implicit format solving the partial differential equations and the method of steps for differential difference equations, we give the numerical simulations to illustrate the theoretical results above.

#### Example 1

**Figure 1:**
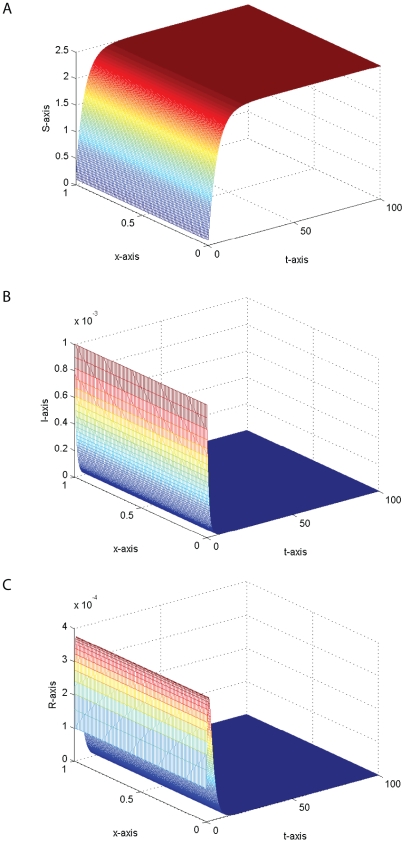
The temporal solution found by numerical integration of problem (0.3) under conditions (0.4) and (0.15) with 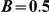, 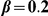, 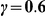, 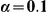, 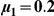, 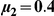, 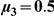, 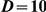, 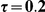 and 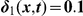, 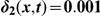, 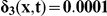, 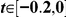.

In system (0.3), let 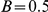, 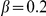, 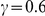, 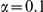, 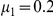, 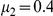, 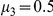, 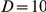 and 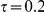. System (0.3) with above coefficients has a unique disease-free steady state 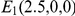. It is easy to show that the basic reproduction number of system (0.3) is 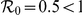. By Theorem 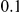 we see that 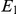 is locally stable. Numerical simulation illustrates our result (see Fig. 1).

#### Example 2

**Figure 2:**
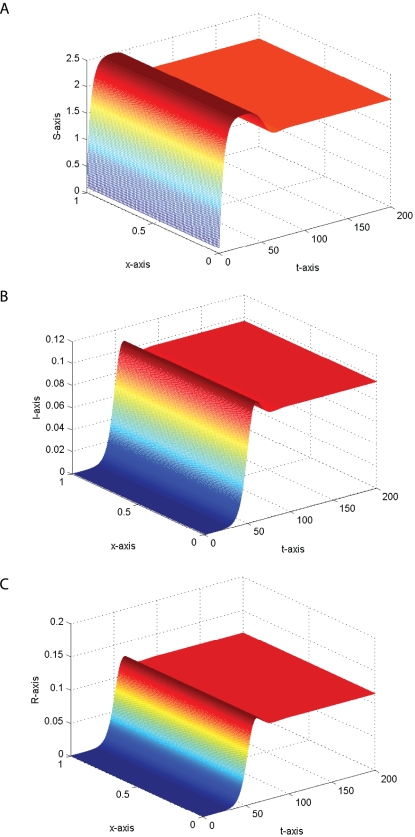
The temporal solution found by numerical integration of problem (0.3) under conditions (0.4) and (0.15) with 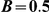, 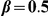, 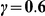, 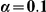, 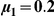, 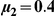, 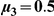, 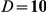, 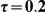 and 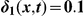, 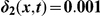, 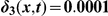, 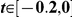.

In system (0.3), we set 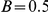, 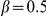, 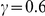, 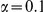, 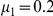, 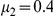, 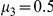, 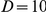 and 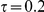. System (0.3) with above coefficients has a disease-free steady state 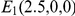 and an endemic steady state 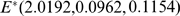. It is easy to show that the basic reproduction number of system (0.3) is 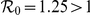. Theorem 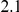 shows that 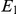 is unstable and 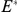 is locally stable. It follows from Theorem 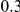 that system (0.3) always has a travelling wave solution with speed 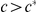 connecting 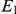 and 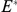. The fact is illustrated by the numerical simulation in Fig. 2. Clearly, Fig. 2 shows that the travelling wave solution does not possess the monotonicity, this seems to be due to the feature of the prey-predator interaction.

## Discussion

In this paper, we have dealt with the existence of travelling wave solutions for an SIR epidemic model with nonlinear incidence rate, spatial diffusion and time delay. At first, by analyzing the corresponding characteristic equations, we discussed the local stability of a disease-free steady state and an endemic steady state to system (0.3) under homogeneous Neumann boundary conditions. We have shown in Theorem 0.1 that time delay and spatial diffusion are negligible for the local stability of the steady states to system (0.3). By using the cross iteration method and the Schauder's fixed point theorem, we reduced the existence of travelling wave solutions to the existence of a pair of upper-lower solutions. By constructing a pair of upper-lower solutions, we derived the existence of a travelling wave solution connecting the uninfected steady state 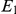 and the endemic steady state 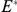.

**Figure 3:**
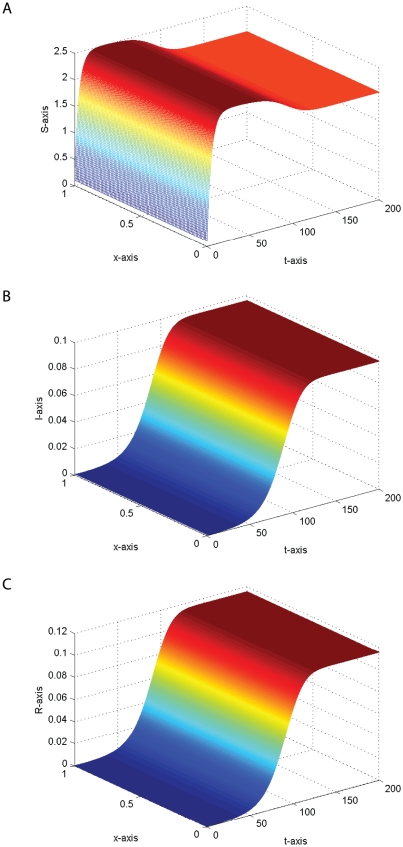
The temporal solution found by numerical integration of problem (0.3) under conditions (0.4) and (0.15) with 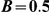, 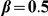, 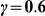, 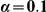, 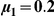, 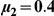, 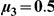, 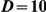, 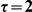 and 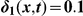, 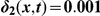, 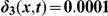, 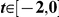.

In fact, we also find that time delay 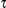 can influence the monotone of the travelling wave solution connecting the disease-free steady state 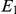 and the endemic steady state 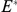. The effect of time delay on the travelling wave solution is illustrated by comparing the numerical simulations in Figs. 2 and 3.
