# Correction: Travelling Waves of a Delayed SIR Epidemic Model with Nonlinear Incidence Rate and Spatial Diffusion

**DOI:** 10.1371/annotation/308a02cb-c4a8-46db-a785-b218f197bba3

**Published:** 2013-08-30

**Authors:** Jing Yang, Siyang Liang, Yi Zhang

The abstract and Introduction section of the article contain overlap in text with that in the publication below:

Gan Q, Xu R, Yang P (2011) Travelling waves of a delayed SIRS epidemic model with spatial diffusion. Nonlinear Analysis: Real World Applications 12:1468-1218.

The authors apologize for this overlap and for omitting to cite the publication by Gan et al. in the article.

Gan et al. studied a SIRS-type epidemic model, in which the incidence rate is bilinear. The SIRS-type epidemic model means that susceptible individuals become infectious, then removed with immunity after recovery from infection and then susceptible again when the temporary immunity fades away. In our article, we employed a nonlinear incidence rate and we dealt with the existence of travelling wave solutions for an SIR epidemic model with nonlinear incidence rate, spatial diffusion and time delay. 

